# 666. Outcomes Using Cefiderocol for the Treatment of *Acinetobacter baumannii* Infections from the PROVE (Real-World Evidence) Study

**DOI:** 10.1093/ofid/ofac492.718

**Published:** 2022-12-15

**Authors:** Stephen Marcella, Emir Kobic, Amy L Carr, Benjamin Georgiades, Caroline Margiotta

**Affiliations:** Shionogi Inc, Florham Park, New Jersey; Banner University Medical Center Phoenix, Phoenix, Arizona; AdventHealth Orlando, Orlando, Florida; Shionogi, Inc, Florham Park, New Jersey; Genesis Research, Hoboken, New Jersey

## Abstract

**Background:**

PROVE is an ongoing international, retrospective study assessing cefiderocol (CFDC) for Gram-negative (GN) infections. Carbapenem-resistant Acinetobacter baumannii (CRAB) infections are difficult-to-treat with limited treatment options. CFDC is a novel sidero cephalosporin with activity against CRAB. This analysis describes the outcomes and treatment patterns of CFDC treatment in CRAB infections from this study.

**Methods:**

Patients were eligible if they received ≥ 72 hours of CFDC. Key patient characteristics, infecting pathogen susceptibility, illness severity, and treatment patterns were assessed. Fourteen and 30-day all-cause mortality (ACM) and clinical cure were examined as outcomes. Susceptibility testing was performed locally. Serious adverse drug reactions (SADR) were recorded.

**Results:**

To date,123 patients treated with CFDC at 12 sites were included. Forty-one had monomicrobial (n=29) or polymicrobial (n=12) *Acinetobacter baumannii (*AB) infection. All but one were CRAB. The median age was 53 years; 71% were male. The most prevalent comorbidity was severe burns (N=9, 22%). Sixty-one percent of patients received CFDC in the ICU, 51% required mechanical ventilation, and 34% required vasopressor support. The median time from positive culture to CFDC initiation was 5 days. CFDC monotherapy was used in 61%. Tetracyclines were the most common concurrent GN antibiotics used with CFDC (N = 8, 19.5%). Targeted therapy with or without prior GN antibiotics was used in 76%, salvage in 20%, and empirical in 2%. Susceptibility results were available for 28 AB cultures from 28 patients of which 82% were susceptible to CFDC. Post-CFDC 14- and 30-day ACM was 12% (95% CI: 4%-26%) and 22% (95% CI: 11%-38%), respectively. Clinical resolution was achieved in 59% (95% CI: 42% -74%). Thirty-day ACM varied by susceptibility to CFDC: 26% for susceptible, 40% for resistant. One SADR (interstitial nephritis) was reported.

Cefiderocol Use Patterns in Acinetobacter baumannii

**Figure:**
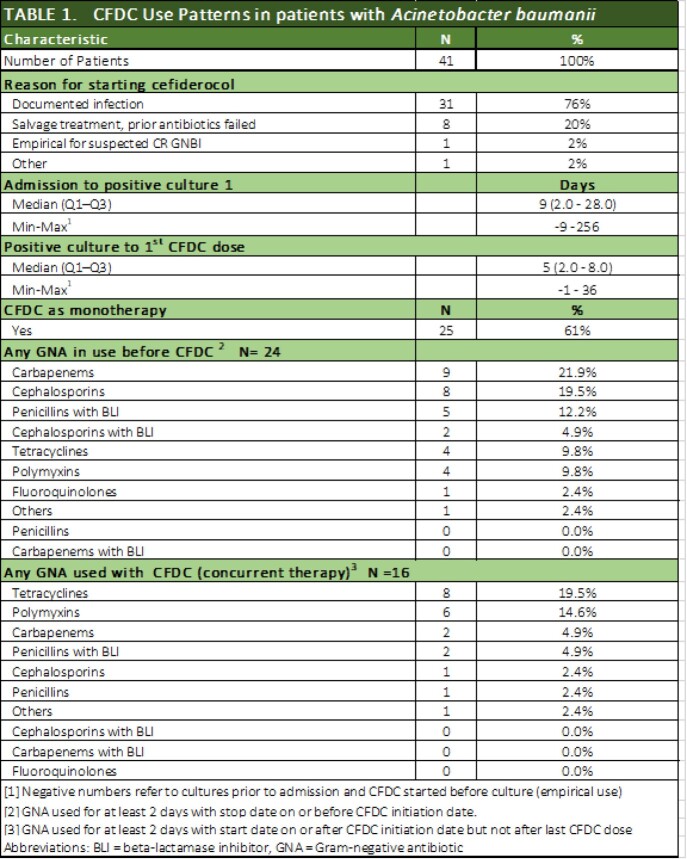

**Figure:**
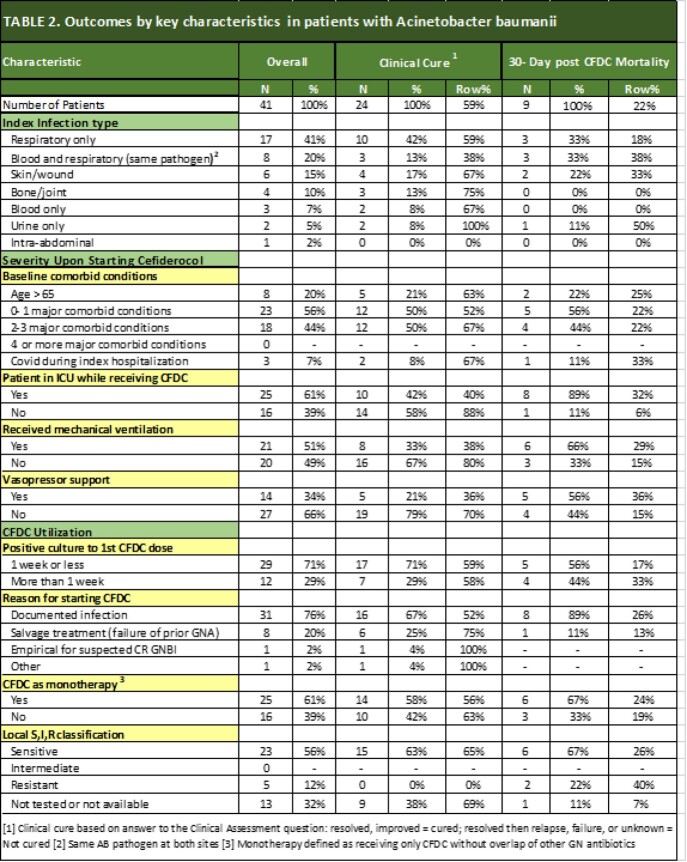

**Conclusion:**

Real-world use of CFDC for AB demonstrates that most patients were complex with multiple comorbidities and severe illness prior to treatment. It was used mostly as targeted therapy. CFDC may be a treatment option in these difficult-to-treat infections.

**Disclosures:**

**Stephen Marcella, MD, MPH**, Shionogi: Shionogi employee|Shionogi, Inc: Employee **Amy L. Carr, PharmD, BCIDP**, Shionogi: Advisory Board **Benjamin Georgiades, PharmD**, Shionogi, Inc: Employee **Caroline Margiotta, MA**, Shionogi, Inc: contracting work for Shionogi, Inc.

